# Predictors for Acceptance of Sexual Aggression Myths Among People Using Cyberporn: Cross-Sectional Study

**DOI:** 10.2196/75485

**Published:** 2025-10-16

**Authors:** Farah Ben Brahim, Germano Vera Cruz, Robert Courtois, Yasser Khazaal

**Affiliations:** 1 Addiction Medicine, Faculty of Biology and Medicine, University of Lausanne, Rue du Bugnon 21, Lausanne, CH-1011, Switzerland, 41 21 692 50 1; 2 QualiPsy UR 1901, Faculty of Arts et Human Sciences, University of Tours, Tours, France; 3 Department of Psychology, UR 7273 CRP-CPO, UFR Human, Social Sciences and Philosophy, University of Picardie Jules Verne, Amiens, France

**Keywords:** compulsive cyberporn, cyberporn, multivariate regression, sexual aggression myths, sexual coercion

## Abstract

**Background:**

Although the acceptance of sexual aggression myths (ASAM) has been intensively studied over the past 30 years, there is still a lack of conclusive information on its relationships with a given set of cognitive and behavioral variables.

**Objective:**

This study aimed to examine to what extent sexual coercion, compulsive cyberporn use (CCU), arousing cyberporn scenes, moral incongruence, impulsivity, and sexual self-esteem can predict the ASAM—assessed with the Acceptance of Modern Myths About Sexual Aggression (AMMSA) score—among people who use cyberporn.

**Methods:**

Overall, 1557 English speakers who used cyberporn at least once during the previous 6 months (mean age 33.3, SD 10.9 years; men: n=1000, 64.2%; women: n=557, 35.8%) were included in this study after completing an online questionnaire. The questionnaire included measures of the AMMSA, sexual coercion experiences including perpetration and victimization, CCU, arousing cyberporn scenes, moral incongruence, impulsivity, and sexual self-esteem. Data analytics included descriptive statistics, Pearson correlation, analysis of variance, and multivariate linear regression.

**Results:**

Male participants had significantly higher scores than their female counterparts regarding the following variables: AMMSA (*t*_1551_=12.46, *P*<.001), sexual violence perpetration (*t*_1551_=3.10, *P*<.001), CCU (*t*_1551_=8.05, *P*=.003), aroused by “groups with several females” porn scenes (*t_1551_*=8.71, *P*=.002), and age (*t_1551_*=7.11, *P*<.001). Female participants had significantly greater mean scores than their male counterparts regarding the following variables: sexual violence victimization (*t*_1551_=−11, *P*<.001), aroused by “submission” pornographic scenes (*t*_1551_=−5, *P*<.001), aroused by “soft” pornographic scenes (*t*_1551_=−2.40, *P*=.03), and aroused by “groups with several males” pornographic scenes (*t*_1551_=−5.93, *P*<.001). Among the male participants, AMMSA scores were significantly predicted by scores of sexual coercion (both perpetration and victimization; β=.042, *P*<.001 and β=.023, *P*=.03, respectively), CCU (*β*=.339, *P*<.001), arousing cyberporn scenes displaying humiliation and groups of males (*β*=.136, *P*=.003), sexual self-esteem (*β*=.164, *P*<.001), and moral incongruence (*β*=.131, *P*<.001). Among the female participants, AMMSA scores were significantly predicted by the scores of sexual coercion perpetration (*β*=.060, *P*<.001), CCU (*β*=.287, *P*<.001), arousing cyberporn scenes with several females (*β*=−.111, *P*=.02), sexual self-esteem (*β*=.188, *P*<.001), and age (*β*=.025, *P*<.001). Age emerged as a distinct factor in women, with older participants more inclined to accept sexual aggression myths.

**Conclusions:**

This study identified key psychological and behavioral correlates of the ASAM across genders. CCU, sexual coercion perpetration, and sexual self-esteem predicted the ASAM. Impulsivity and moral incongruence were salient among men, and age was salient among women. Gender differences emerged in arousal of pornographic content.

## Introduction

Sexual aggression myths [1], including rape myths [2], are biased, stereotyped, or false beliefs about sexual aggression, survivors [3], and sexual offenders that create an adverse atmosphere for survivors, including justifications for coercion, victim blaming, and gendered power dynamics [4,5]. Those beliefs affect how people interact with survivors, especially whether they believe them or blame them for their abuse [1,6]. Such beliefs are strong barriers to the dissemination of asking for sexual consent [7] and to the prevention of all kinds of sexual aggression [8]. Among others, deeply rooted sex, gender, and social stereotypes are associated with the acceptance of sexual aggression myths (ASAM) [2,4,5,9], especially among men [10]. A questionnaire related to the Acceptance of Modern Myths About Sexual Aggression (AMMSA) [1] conceptualizes such myths about sexual aggression, including subtle and still “socially acceptable” beliefs that downplay or justify sexual violence, not limited to rape in the legal sense. These myths are part of broader cultural dynamics and media narratives, which warrant further exploration.

In addition to distorting the reality of sexual violence, these myths reinforce negative cultural stereotypes [3] that uphold male sexual dominance and minimize or excuse acts of aggression. Payne et al’s [3] approach draws attention to the common social beliefs that support the persistence and propagation of these myths, emphasizing that the ASAM is part of a broader cultural narrative. Such narratives are partly vehicled by media, including some porn content [11]. Given these societal impacts, researchers have explored how media, particularly pornography, are associated with the ASAM. In this context, the relationship between sexual aggression myths and porn use has been particularly examined.

For instance, an earlier meta-analysis concluded that pornography is associated with the ASAM in experimental research but not in nonexperimental studies [12]. A more recent meta-analysis by Hedrick [13] showed that porn, especially porn involving violent content, is positively associated with the ASAM. Borgogna et al [14] indicated, however, that hostile sexism positively predicts the ASAM regardless of gender or porn use. Possibly, the conflicting results arise from cultural differences, differences in porn content, and violence-related porn materials classifications [15], as well as possible individual differences, including variations in the way people are using porn or cyberporn (internet porn use) [16-19] and in compulsive cyberporn use (CCU) [16,17]; a behavioral marker of problematic engagement in porn—rather than frequency alone [20,21]—may better account for these inconsistencies. Of note, CCU is characterized by an increase in pornography use, accompanied by a loss of control and associated negative consequences [22-26]. However, studies directly examining the link between CCU and ASAM are still limited (eg, [16]). This highlights the need to examine how individual differences intersect with both CCU and the ASAM.

Individual differences among variables previously linked to CCU—such as pornography-related moral incongruence [27], impulsivity [28,29], and sexual self-esteem [16]—may help explain some possible relationship with the ASAM. One such factor, moral incongruence, refers to the psychological distress that arises when pornography use conflicts with an individual’s internalized moral values [27,30]. To reduce this tension, some may adopt rationalizations or belief systems—such as the ASAM—that serve to legitimize or minimize the moral implications of their behavior. As such, porn-related moral incongruence may not only be a correlate of CCU but also a psychological mechanism contributing to the endorsement of the ASAM. Impulsivity, a neuropsychological trait marked by a tendency for spontaneous actions without careful consideration, has been positively associated with the ASAM [31], suggesting that individuals with higher impulsivity may be more inclined to rationalize or justify sexual violence due to limited self-control and reflection on their actions. Similarly, sexual self-esteem, which has shown a modest yet positive association with sexual coercion [16,32], refers to the individual’s perception of their own sexual value, confidence, and competence in sexual contexts [33]. It has been associated with sexual satisfaction, relational dynamics, and vulnerability to coercion-related beliefs and behaviors [34,35], making it relevant in the study of the ASAM. The link between sexual self-esteem and sexual coercion may be particularly relevant for people with cyberporn use, as pornography could shape or reinforce perceptions of sexual worth and attitudes toward sexual behaviors [6].

In addition to these individual factors, past personal experiences may interfere with beliefs about sexual aggression. Thus, personal experiences related to sexual coercion [36-38] may shape possible associations between CCU and the ASAM, given the documented links between prior sexual coercion experiences and the ASAM [39].

Another factor that may help to explain these associations is the type of pornographic content that arouses users. This neglected dimension that may contribute to such an association between porn use and the ASAM is the arousal that people may have in relation to specific porn contents [40,41] (ie, differences may exist between people reporting arousal from humiliation scenes and those reporting arousal from soft ones). Such porn scripts may influence, or reciprocally interact, with people’s beliefs about gender roles and sexual conduct, especially when they portray themes of control, dominance, or coercion [42-44]. By normalizing aggressive sexual relations and minimizing the value of consent, these scripts have the potential to reinforce the ASAM [42-44].

Bringing these threads together, this study aimed to explore the relationships between the above-discussed variables and the ASAM. Specifically, it aimed at exploring, by gender group (male vs female), the relationships between a set of variables related to the participants’ sexual coercion experiences, CCU, arousing cyberporn scenes, moral incongruence, impulsivity, sexual self-esteem, and ASAM—assessed with the AMMSA score—among people who use cyberporn.

## Methods

### Design of the Study

In order to achieve the aim, the study assessed a relatively large sample of participants (when compared with previous studies, eg, [2,36-38]) who used cyberporn at least once during the previous 6 months.

No precise hypotheses were formulated before data collection, as the investigation was exploratory and data-driven. Exploratory research permits adaptation and exploration of new pathways or new data analysis methodologies as the study progresses, in contrast to structured research with preconceived assumptions. This approach encourages the use of different analytical methods for extracting information from data without being constrained by particular ideas or by following the path carved by previous research [45]. This has the benefit of opening up novel opportunities for investigation and avoiding bias or errors from earlier research or established theories [45].

### Participants

Participants were recruited through Prolific, a crowdsourcing platform widely used for academic research [46,47]. The study was conducted entirely online using the SphinxOnline platform. Eligible participants were English-speaking adults (aged 18 y or older) who reported having viewed pornography at least once during the past 6 months. After accessing the survey link, participants were presented with a detailed information sheet and eligibility questions. Only those who met the inclusion criteria were granted access to the full questionnaire.

### Materials

#### Criterion Variables

##### Acceptance of Sexual Aggression Myths

Gerger et al’s [1] 30-item AMMSA was shortened [48] to measure the ASAM (Multimedia Appendix 1; the measures are available for consultation if needed). This 11-item (7-point scale from “I totally disagree” to “I totally agree”) assesses participants’ tolerance for the ASAM and female sexual aggression (eg, “When a woman starts a relationship with a man, she should be aware that the man will assert his right to have sex”; “Many women tend to exaggerate the problem of male violence”). Each participant has an overall rating. Higher scores indicate greater acceptance of myths. The Cronbach α scale was 0.94 in this study.

This questionnaire has been validated and implemented in numerous countries; it is, in fact, one of the most frequently employed measures in this field [1,48,49].

### Predictor Variables 

#### Demographic Data

Sex, age, sexual orientation, and relationship status were the demographic details provided by the participants.

#### Violent and Coercive Sexuality

The Sexual Experience Survey (SES) assessed sexual coercion experiences of perpetration and victimization in youths over the age of 14 years [50,51]. Victimization (eg, touching, kissing, rape) was determined by the 11-item victimization form. Perpetration (eg, touching, kissing, rape) was determined by the 11-item perpetration form. We determined the total perpetration and victimization scores of each participant for this research. The subscales’ α values were 0.91 and 0.93, respectively.

#### Compulsive Cyberporn Use

We used the 8-item short form of the Compulsive Internet Use Scale (CIUS) to measure CCU [52,53]. Previous research [54,55] adapted the CIUS for cybersex, while this study adapted the CIUS for cyberporn, following previous methods [16,17], with the word “Internet” signifying pornographic websites. This unidimensional short form is internally consistent [52]. A 5-point scale (from “Never” to “Very often”) tested CCU, with higher values indicating higher CCU. In this study, this scale’s α was 0.90.

#### Arousing Cyberporn Scenes

We assessed 5 cyberporn scenes by asking about the arousal level toward these scenes (submission, humiliation, soft porn, groups with several males, and groups with several females), using the following instruction: “The following pornographic scenes are:”. We used a 4-point scale ranging from “Very arousing” to “Not arousing at all.”

#### Moral Incongruence

We relied on Grubbs et al’s [27] item “I believe that pornography use is morally wrong” to assess pornography’s negative moral perception with a 7-point scale (“Strongly disagree” to “Strongly agree”).

#### Impulsivity

It was assessed using the Short Urgency, Premeditation, Perseverance, Sensation Seeking, Positive Urgency (UPPS-P) Impulsive Behavior Scale [56,57]. Specifically, the two features most frequently associated with addictive behaviors, positive and negative urgency (4-point response scale from “Agree strongly” to “Disagree strongly”) [58-60], were assessed using only 8 of the measure’s 20 items; these 8 items are related to two dimensions: positive urgency (measures the tendency to act impulsively due to positive affect; eg, “When I’m really excited, I tend not to think about the consequences of my actions”) and negative urgency (measures the tendency to act impulsively due to negative affect; eg, “When I am upset, I often act without thinking”). Two scores were awarded to each participant. Before calculating the two scores, the scale items were reverse-coded. Impulsivity increased with higher scores. The subscales’ α values were 0.79 and 0.82 for positive and negative urgency, respectively.

#### Sexual Self-Esteem

Based on the self-esteem single-item scale [61], we rated the sexual self-esteem of the participants with the following derived item: “I have high sexual self-esteem” on a 4-point scale from “Not very true of me” to “Very true of me.”

### Ethical Considerations

The study relied on an anonymous SphinxOnline survey. The Research Ethics Committee of Tours-Poitiers in France (2020-04-05) approved the study. All procedures were conducted in accordance with the Declaration of Helsinki and relevant institutional guidelines. Participants provided informed consent online before participation, after being fully informed about the study’s aims, procedures, anonymity, and their right to withdraw at any time without consequences. No personally identifying data were collected. Data were collected, stored, and analyzed anonymously. Participants were recruited through Prolific, a platform widely used for ethical participant recruitment in academic research [46,47]. Inclusion criteria included being at least 18 years old and having watched pornographic material at least once in the past 6 months. The survey took approximately 15 minutes to complete. The participants received time compensation for their study participation via the Prolific platform (approximately £10 [approximately US $14] per hour of participation). This study was not registered on a public trial registry; however, the protocol was approved in advance by the ethics committee, and no deviations from the original procedures occurred. No adverse events were reported during data collection.

### Reporting

This study follows the recommendations of the STROBE (Strengthening the Reporting of Observational Studies in Epidemiology) guidelines for reporting observational research [62]. Although STROBE was initially developed for epidemiological studies, it is broadly applicable to cross-sectional observational designs in psychology and social sciences. We ensured transparency and completeness in reporting, in line with the EQUATOR Network recommendations [62].

### Statistical Analysis

It must be noted that the data had 0.2%-0.7% of missing values in the sociodemographic variables.

First, we conducted descriptive analyses on the study’s predictor and criterion variables. These analyses were conducted separately on male versus female participants.

Second, we built a correlation matrix including all the study variables.

Third, we tested the normality of the dependent variable (AMMSA score) using the Shapiro-Wilk test for normality. The test was significant (*W*=0.878, *df*=1557; *P*<.001), indicating that the distribution of this variable was significantly different from a normal distribution [63]. In addition, we conducted multicollinearity tests on the independent variables. The results indicated no significant multicollinearity.

Fourth, and given that the assumption of normality had not been met, we built two robust multivariate linear regression models (one per gender group). These linear regressions used the above predictor variables (except for sexual orientation and relationship status) to predict AMMSA scores, the study’s criterion variable. Robust linear regression can be used to improve the accuracy of predictions when the residuals are nonnormal and when the data contain outliers [64].

Finally, we conducted an ANOVA to test the effect of sociodemographic variables (sexual orientation and relationship status) on the AMMSA score.

All statistical analyses were conducted using the software SPSS Statistics version 29 (IBM Corp), except for the regression analyses, which were conducted using the R package “robustbase” [65]. These analyses were conducted with different statistical software that have different ways of dealing with missing values (eg, SPSS allows analysis considering only the participants without any missing values in each variable; on the contrary, the algorithm used for regression requires missing values imputation using the nearest neighbor approach).

## Results

### Descriptive Statistics and Inferential Comparison

Overall, 1590 English-speaking individuals fully completed the online questionnaire. However, 1557 participants were included in this study, among them 64.2% (n=1000) were men, and 35.8% (n=557) were women. The age of participants ranged from 18 to 75 (mean 33.3, SD 10.9) years. During the study period, 497 (31.9%) participants were single; 663 (42.6%) were in a relationship, not married; 389 (25%) were in a relationship, married; and 8 (0.5%) were widows. The participants’ sexual orientation distribution was as follows: heterosexual 1228 (78.9%), homosexual 98 (6.3%), bisexual 200 (12.8%), and other 31 (2%). It must be noted that, given this study’s objective, outlined above, 33 participants were excluded; among them, 27 were nonbinary, and 6 did not indicate their gender. Participants’ countries of residence can be found in Multimedia Appendix 2.

Table 1 presents the descriptive statistics of all variables by gender (male vs female participants) and the respective mean comparison.

**Table 1.** Descriptive statistics (by gender: male n=998 vs female n=555) of the continuous and the ordinal variables included in this study design to predict the acceptance of sexual aggression myths in a multicountry sample.

Variable categories/variables	Scale/range	Mean male	SD male	Mean female	SD female	*t* test (*df*)	*P* value
Criterion variable							
AMMSA^a^ acceptance of sexual aggression myths (total score)	1‐7	3.08	1.32	2.25	1.20	12.46 (1551)	<.001^b^
Predictor variables							
Violent and coercive sexuality (2 variables)							
SES-P^c^ (total score)	0‐77	1.63	6.68	.81	3.78	3.10 (1551)	<.001^b^
SES-V^d^ (total score)	0‐105	1.37	4.50	9.09	16.19	−11 (1551)	<.001^b^
CCU^e^ (1 variable)							
CIUS^f^ adapted to cyberporn use (total score)	1‐5	2.58	0.88	2.18	0.97	8.05 (1551)	.003^b^
Arousing cyberporn scenes (5 variables)							
Aroused by “submission” pornographic scenes	1‐4	2.51	1.05	2.77	0.98	−5 (1551)	<.001^b^
Aroused by “humiliation” pornographic scenes** **	1‐4	1.74	1.01	1.66	1.02	1.39 (1551)	.83
Aroused by “soft” pornographic scenes	1‐4	2.62	0.91	2.75	0.99	−2.40 (1551)	.02^g^
Aroused by “groups with several males” pornographic scenes	1‐4	1.83	1.05	2.18	1.13	−5.93 (1551)	<.001^b^
Aroused by “groups with several females” porn scenes** **	1‐4	2.83	1.04	2.34	1.08	8.71 (1551)	.002^b^
Impulsivity (2 variables)							
UPPS-P^h^ negative urgency impulsivity	1‐4	2.63	0.65	2.40	0.65	6.61 (1551)	.89
UPPS-P positive urgency impulsivity	1‐4	2.55	0.58	2.42	0.60	4.33 (1551)	.18
Sexual self-esteem (1 variable)							
Sexual self-esteem	1‐4	2.35	0.87	2.30	0.94	1.09 (1551)	.22
Moral incongruence (1 variable)							
Pornography negative moral perception	1‐7	2.21	1.62	2.30	1.57	−1.14 (1551)	.75
Sociodemographics (1 variable)							
Age (years)	18‐75	34.67	11.45	30.87	9.26	7.11 (1551)	<.001^b^

a AMMSA: Acceptance of Modern Myths About Sexual Aggression.

b Significant at <.01.

c SES-P: Sexual Experience Survey–Perpetration.

d SES-V: Sexual Experience Survey–Victimization.

e CCU: compulsive cyberporn use.

f CIUS: Compulsive Internet Use Scale.

g Significant at <.05.

h UPPS-P: Urgency, Premeditation, Perseverance, Sensation Seeking, Positive Urgency.

As can be seen in this table, male participants had significantly higher scores than their female counterparts regarding the following variables: ASAM (AMMSA total score), SES–Perpetration (total score), CCU (CIUS total score), aroused by “groups with several females” porn scenes, and age. Female participants had significantly greater mean scores than their male counterparts on the following variables: SES–Victimization (total score), aroused by “submission” pornographic scenes, aroused by “soft” pornographic scenes, and aroused by “groups with several males” pornographic scenes.

### All Variables Correlation Matrix

Table 2 shows the correlation coefficients (by gender) between the variables included in the study, two-by-two combinations. As shown in these two tables, the strongest correlations are (*r*≥0.20) between AMMSA and SES–Perpetration (+), AMMSA and CCU (+), SES–Perpetration and SES–Victimization (+), humiliation porn scenes and submission porn scenes (+), soft porn scenes and humiliation porn scenes (−), porn scenes with group of males and humiliation (+), UPPS-P negative and positive urgency and CCU (+), and age and submission porn scenes (−) with a far more important association among women than men.

**Table 2.** Correlation (by gender: male n=998 vs female n=555) between the variables included in this study design to predict the acceptance of sexual aggression myths in a multicountry sample.

Variables by gender	AMMSA^a^ (total score)	SES-P^b^ (total score)	SES-V^c^ (total score)	CIUS^d^ adapted to cyberporn use (total score)	Aroused by “submission” pornographic scenes	Aroused by “humiliation” pornographic scenes	Aroused by “soft” pornographic scenes	Aroused by “groups with several males” pornographic scenes	Aroused by “groups with several females” porn scenes	UPPS-P^e^ negative urgency impulsivity	UPPS-P positive urgency impulsivity	Sexual self-esteem	Pornography negative moral perception	Age
AMMSA (total score), *r*														
Male participants	—^f^													
Female participants	—													
SES-P (total score), *r*														
Male participants	0.235^g^	—												
Female participants	0.217^g^	—												
SES-V (total score), *r*														
Male participants	0.076^h^	0.556^g^	—											
Female participants	0.017	0.248^g^	—											
CIUS adapted to cyberporn use (total score), *r*														
Male participants	0.271^g^	0.093^g^	0.083^g^	—										
Female participants	0.247^g^	0.127^g^	0.081	—										
Aroused by “submission” pornographic scenes, *r*														
Male participants	0.124^g^	0.032	0.050	0.174^g^	—									
* *Female participants	0.052	0.033	0.069	0.123^g^	—									
Aroused by “humiliation” pornographic scenes, *r*														
Male participants	0.144^g^	0.088^g^	0.054	0.121^g^	0.420^g^	—								
Female participants	0.053	0.073	0.105^h^	0.073	0.341^g^	—								
Aroused by “soft” pornographic scenes, *r*														
Male participants	−0.012	0.013	−0.032	0.090^g^	−0.058	−0.215^g^	—							
Female participants	−0.013	0.088^h^	−0.024	0.094^h^	−0.009	−0.168^g^	—							
Aroused by “groups with several males” pornographic scenes, *r*														
Male participants	−0.008	0.045	0.092^g^	0.078^h^	0.164^g^	0.366^g^	−0.145^g^	—						
Female participants	0.029	0.025	0.009	0.122^g^	0.254^g^	−0.168^g^	−0.199^g^	—						
Aroused by “groups with several females” porn scenes, *r*														
Male participants	0.030	0.036	−0.038	0.047	0.006	−0.061	0.092^g^	−0.113^g^	—					
Female participants	−0.056	0.042	0.017	0.067	0.152^g^	0.188^g^	−0.086^h^	0.267^g^	—					
UPPS-P negative urgency impulsivity, *r*														
Male participants	−0.144^g^	−0.152^g^	−0.068^h^	−0.223^g^	−0.085^g^	−0.079^h^	0.016	−0.028	−0.015	—				
Female Participants	−0.104^h^	−0.066	−0.015	−0.228^g^	−0.100^h^	−0.144^g^	0.065	−0.065	−0.122^g^	—				
UPPS-P positive urgency impulsivity, *r*														
Male participants	−0.120^g^	−0.116^g^	−0.041	−0.233^g^	−0.129^g^	−0.078^h^	−0.030	−0.020	−0.064^h^	0.606^g^	—			
Female participants	−0.078	−0.144^g^	−0.120^h^	−0.276^g^	−0.102^h^	−0.072	0.002	−0.053	−0.177^g^	0.565^g^	—			
Sexual self-esteem, *r*														
Male participants	0.086^g^	0.081^h^	0.031	−0.090^g^	0.005	−0.040	−0.013	−0.030	0.030	0.214^g^	0.108^g^	—		
Female participants	0.164^g^	0.064	0.044	0.005	−0.024	−0.034	0.065	−0.013	0.013	0.096^h^	0.005	—		
Pornography negative moral perception, *r*														
Male participants	0.202^g^	0.097^g^	0.044	0.120^g^	−0.010	−0.010	0.074^h^	−0.047	0.019	−0.006	−0.054	−0.004	—	
Female participants	0.082	0.084^h^	−0.030	0.088^h^	−0.027	−0.044	0.148^g^	−0.171	−0.097^h^	0.008	−0.023	−0.030	—	
Age, *r*														
Male participants	−0.037	0.051	−0.006	−0.232^g^	−0.076^h^	−0.076^h^	−0.062	0.005	−0.025	0.084^g^	0.128^g^	0.079^h^	−0.075^h^	—
Female participants	0.152	0.015^g^	0.071	−0.118^g^	−0.201^g^	−0.067	−0.105^h^	0.043	0.004	0.090^h^	0.195^g^	0.124^g^	−0.123^g^	—

a AMMSA: Acceptance of Modern Myths About Sexual Aggression.

b SES-P: Sexual Experience Survey–Perpetration.

c SES-V: Sexual Experience Survey–Victimization.

d CIUS: Compulsive Internet Use Scale.

e UPPS-P: Urgency, Premeditation, Perseverance, Sensation Seeking, Positive Urgency.

f Not applicable.

g *P*<.001.

h *P*<.05.

### Associations Statistics

Table 3 (regression model 1, *R*^2^ [explained variance]=0.42, male participants; regression model 2, *R*^2^=0.41, female participants) displays the relationships between the independent variables and the dependent variable, resulting from the multivariate regression analyses.

**Table 3.** Multivariate associations (by gender: male n=1000 vs female n=557) between the predictor variables and the criterion variable (Acceptance of Modern Myths About Sexual Aggression total score) included in this study design to predict the acceptance of sexual aggression myths in a multicountry sample.

Variables by gender	*β* (95% CI)	Error	*P* value^a^	VIF^b^
Violent and coercive sexuality (2 variables)				
SES-P^c^ (total score)				
Male participants	.042 (0.028–0.056)	0.007	<.001	1.53
Female participants	.060 (0.034–0.086)	0.013	<.001	1.11
SES-V^d^ (total score)				
Male participants	−.023 (−0.043 to −0.003)	0.010	.03	1.47
Female participants	−.006 (−0.012 to 0.000)	0.003	.07	1.11
CCU^e^ (1 variable)				
CIUS^f^ adapted to cyberporn use (total score)				
Male participants	.339 (0.246–0.431)	0.047	<.001	1.17
Female participants	.287 (0.185–0.389)	0.052	<.001	1.15
Arousing cyberporn scenes (5 variables)				
Aroused by “submission” pornographic scenes				
Male participants	.059 (−0.022 to 0.139)	0.041	.15	1.28
Female participants	.080 (−0.024 to 0.184)	0.053	.13	1.22
Aroused by “humiliation” pornographic scenes				
Male participants	.136 (0.047–0.224)	0.045	.003	1.42
Female participants	.040 (−0.063 to 0.143)	0.053	.45	1.30
Aroused by “soft” pornographic scenes				
Male participants	−.044 (−0.129 to 0.042)	0.044	.32	1.08
Female participants	−.065 (−0.163 to 0.033)	0.050	.19	1.11
Aroused by “groups with several males” pornographic scenes				
Male participants	−.087 (−0.164 to −0.010)	0.039	.03	1.18
Female participants	−.017 (−0.110 to 0.077)	0.047	.73	1.31
Aroused by “groups with several females” porn scenes				
Male participants	.004 (−0.069–0.077)	0.037	.92	1.03
Female participants	−.111 (−0.202 to −0.020)	0.046	.02	1.12
Impulsivity (2 variables)				
UPPS-P^g^ negative urgency impulsivity				
Male participants	.180 (−0.328 to −0.032)	0.075	.02	1.68
Female participants	.123 (−0.298 to 0.051)	0.089	.17	1.54
UPPS-P positive urgency impulsivity				
Male participants	−.031 (−0.133 to 0.196)	0.084	.71	1.63
Female participants	.007 (−0.201 to 0.187)	0.099	.94	1.64
Sexual self-esteem (1 variable)				
Sexual self-esteem				
Male participants	.164 (0.076–0.253)	0.045	<.001	1.07
Female participants	.188 (0.088–0.288)	0.051	<.001	1.04
Moral incongruence (1 variable)				
Pornography negative moral perception				
Male participants	.131 (0.084–0.178)	0.024	<.001	1.03
Female participants	.054 (−0.007–0.114)	0.031	.08	1.07
Sociodemographics (1 variable)				
Age				
Male participants	.003 (−0.003 to 0.010)	0.004	.33	1.11
Female participants	.025 (0.014–0.035)	0.005	<.001	1.15

a *P* value (significant at <.05).

b VIF: variance inflation factors; it is a test of multicollinearity (a value of 1 indicates that there is no multicollinearity; VIFs between 1 and 5 suggest that there is a moderate multicollinearity, but it is not severe enough to warrant corrective measures; VIFs higher than 5 represent critical levels of multicollinearity, indicating that there is a need for corrective measures).

c SES-P: Sexual Experience Survey–Perpetration.

d SES-V: Sexual Experience Survey–Victimization.

e CCU: compulsive cyberporn use.

f CIUS: Compulsive Internet Use Scale.

g UPPS-P: Urgency, Premeditation, Perseverance, Sensation Seeking, Positive Urgency.

The results from model 1 suggest that male participants were more likely to endorse the ASAM if they had higher scores of sexual coercion perpetration, CCU, arousal with pornographic scenes displaying humiliation, negative urgency impulsivity, sexual self-esteem, and moral incongruence. They were less likely to endorse the ASAM if they had higher scores of sexual coercion victimization and arousal toward pornographic scenes displaying groups of several males.

The findings from model 2 suggest that female participants were more likely to endorse the ASAM if they had higher scores of sexual coercion perpetration, CCU, sexual self-esteem, and older age. They were less likely to endorse the ASAM if they had higher scores of arousal toward pornographic scenes displaying groups of several females.

In addition, the ANOVA results show that among male participants, there was no statistical difference between the AMMSA mean score of single (n=338) and in-relationship (n=650) participants. On the contrary, the AMMSA mean scores of heterosexual (n=841), homosexual (n=61), and bisexual (n=92) participants differ statistically (3.14, SD 1.31; 2.84, SD 1.42; 2.75, SD 1.29, respectively; *F*_3,991_=3.81, *P*=.02, η^2^p=0.008). In particular, the AMMSA mean score of heterosexual participants is statistically higher than the AMMSA mean score of bisexual participants (*t*_931_=2.44, *P*=.04). There was no difference between the AMMSA mean score of heterosexual versus homosexual and homosexual versus bisexual participants (*t*_900_=1.56, *P*=.26; *t*_151_=1.21, *P*=.45, respectively).

Among female participants, there was no statistical difference between the AMMSA mean score of single (n=143) and in-couple (n=391) participants. On the contrary, the AMMSA mean scores of heterosexual (n=387), homosexual (n=37), and bisexual (n=112) participants differ statistically (2.47, SD 0.23; 2.02, SD 1.21; 1.74, SD 0.89, respectively; *F*_3,533_=12.48, *P*<.001, η^2^p=0.045. In particular, the AMMSA mean score of heterosexual participants is statistically higher than the AMMSA mean score of bisexual participants (*t*_497_=4.92, *P*<.001). There was no difference between the AMMSA mean score of heterosexual versus homosexual and homosexual versus bisexual participants (*t*_422_=1.56, *P*=.26; *t*_147_=1.21, *P*=.45, respectively).

## Discussion

The two robust multivariate linear regression models highlighted the complex interplay of factors that contribute to the endorsement of the ASAM among male and female participants. The acceptance of these myths was assessed in this study with the AMMSA scale [1], which includes modern and socially embedded myths that justify or downplay a wide range of aggressive sexual behaviors—not limited to rape in its legal definition.

### Principal Results and Comparison With Prior Work

#### Relationships Between Predictors and the Outcome: Male Participants

In the male cohort, as delineated by regression model 1, a multifaceted relationship was observed. Male participants who have perpetrated sexual coercion or have higher CCU scores displayed a greater propensity to endorse sexual aggression myths. This association is further compounded by heightened arousal to specific pornographic content featuring humiliation, indicative of a disturbing association between sexual aggression myths endorsement and the consumption of such specific pornographic content. Interestingly, arousal to scenes involving multiple males correlates with a reduced tendency to endorse sexual aggression myths. The association between arousal to scenes involving multiple males and the AMMSA scores is, however, weak. As such, any interpretations regarding their impact on broader understandings of sexual relationships should be considered tentative and approached with caution. Moreover, it is important to consider the role of the participants’ sexual orientation in this association. Individuals with different sexual preferences might interpret and respond to such content in varied ways, which could further contribute to the complex and nuanced understanding observed in this study.

The significant relationship between the male participants’ negative urgency impulsivity, sexual self-esteem (both positively correlated with sexual aggression myths), and their AMMSA scores found in this study underlines the cognitive and emotional dimensions that fuel these beliefs. These two dimensions suggest the existence of a dual mechanism—emotional dysregulation (via urgency) and cognitive self-enhancement (via sexual self-esteem)—through which individuals may be more susceptible to endorsing harmful myths surrounding sexual aggression. In particular, negative urgency may contribute to justifying coercive behaviors as a way to manage internal tension or frustration, aligning with myths that minimize the responsibility of the aggressor, while sexual self-esteem may support rationalizations of aggression when inflated or contingent on dominance.

The findings from this study also highlighted moral incongruence as an important factor predicting male participants’ AMMSA scores, hinting at an internal dissonance that may perpetuate harmful stereotypes. This particular finding is consistent with our initial hypothesis that moral incongruence would be positively associated with the ASAM, particularly given its established role in compulsive pornography use [66]. Furthermore, it highlights the role of internal psychological conflict—particularly when one’s behaviors (eg, compulsive pornography use) are at odds with personal moral standards. One may hypothesize that this dissonance may lead to defensive cognitive strategies (ie, rationalization of morally conflicting behaviors [67]), such as endorsing rape myths or minimization of aggression, to alleviate guilt or justify behavior [68].

#### Relationships Between Predictors and the Outcome: Female Participants

Conversely, regression model 2 reveals that among female participants, patterns emerged that mirror some aspects of their male counterparts. For instance, female participants with higher scores of perpetration of sexual coercion and those with higher CCU scores are more inclined to validate sexual aggression myths. Notably, the results of this study showed that arousal to pornographic scenarios involving multiple females inversely predicts sexual aggression myths endorsement (negative relationship). Additionally, sexual self-esteem once again appears as a positive predictor—female participants with higher scores of sexual self-esteem are more likely to endorse sexual aggression myths. Age emerges as a factor unique among women, with older female participants being more likely to endorse these myths, potentially reflecting generational shifts in attitudes toward sexual violence. These findings collectively highlight the need for gender-sensitive interventions that address the roots of the ASAM, emphasizing the multifaceted and gender-specific factors that underpin this social issue.

#### Relationships Between Predictors and the Outcome: Male and Female Participants

When comparing male and female participants, common features explained the endorsement of sexual aggression myths, including higher CCU, sexual self-esteem, and perpetration of sexual coercion. All these factors are significantly and positively related to the participants’ AMMSA scores for both sexes. A unique finding in the female cohort was the influence of age, with older women showing a higher likelihood of endorsing sexual aggression myths. The phenomenon was not observed in the male group, highlighting the possible asymmetric evolution of sexual aggression myths across gender. For both groups, arousal from porn scenes involving several people of the same sex was associated with a lower level of the ASAM. More refinement in assessing these results may be useful to understand this apparent paradox. For male participants only, arousing humiliation scenes were associated with higher endorsement of sexual aggression myths. In addition, our findings, like in Worthen’s [69] study, revealed that people with heterosexual orientation have statistically higher AMMSA scores compared to the participants with a bisexual orientation. Similarly, complementary results [70] found that people identifying themselves as queer exhibit lower ASAM levels compared to people with homosexual orientation. These findings suggest that sexual minorities are generally less accepting of sexual aggression myths.

#### Relationships Between All the Variables

One of the notable correlations was between the sexual perpetration scores and the sexual victimization scores among men and women. This association was stronger in men. Research suggests that there can be an overlap between individuals who perpetrate sexual violence and those who are victimized, indicating that these experiences may be interconnected cyclically. People may be both perpetrators and victims of sexual violence, especially in intimate partner violence contexts. Psychological intimate partner violence seems to have an important perpetration-victim overlap [71]. In a technology-facilitated sexual violence context, victimization and perpetration are also linked, especially among adolescent males. Victimization may lead to perpetration as retaliation or learned behavior [72].

Age and arousal to submission pornographic scenes were negatively associated among both men and women. This association was particularly weak in men. This suggests that submissive porn scenarios may be more arousing for younger women compared to older women. According to the results of this study, as women age, they appear to lose interest in submission porn, which may reflect a shift in their sexual preferences. Consequently, questions could be raised about how their interest in submissive real-life sexual behavior evolves in relation to their porn use.

#### Theoretical Implications

Our findings indicate that both individual and societal factors significantly predict the ASAM. The associations observed between CCU, sexual self-esteem, and the ASAM across genders suggest that psychological traits and exposure to pornography-related activities are significantly related to the endorsement of sexual aggression myths. These results extend Payne et al’s [3] conception of sexual aggression myths, which emphasized their cultural foundations, by highlighting the importance of individual-level characteristics—such as impulsivity and moral incongruence—which were found to be associated with the ASAM differently according to gender.

The results illustrate the complex nature of sexual content consumption and sexual identity, therefore extending ASAM theory. According to Worthen [69], people from sexual minorities critique sexual aggression myths more due to discrimination. This study’s findings on pornography use complement and extend this understanding. The multiple associations between a variety of pornographic content and the ASAM (eg, the negative association with same-sex group scenes vs the positive association with humiliation content) suggest that individual traits and content type may moderate the relationship between sexual content exposure and sexual aggression myths. If true, this questions fundamental theoretical paradigms that see porn use as either consistently negative or positive and offers an elaborate theoretical model that can include viewer traits and content-specific effects.

The positive correlation between sexual self-esteem and the ASAM presents a complex and somewhat paradoxical finding. While self-esteem is generally considered protective, higher levels of sexual self-esteem, when combined with certain sexual behaviors (such as compulsive cyberporn consumption), may instead reinforce harmful stereotypes and myths surrounding sexual violence. This may indicate that the ASAM is not merely a product of static cultural beliefs but is dynamically shaped by personal experiences, psychological traits, and behaviors, with distinct gender-specific pathways.

To fully understand the mechanisms surrounding sexual aggression myth endorsement, future theoretical frameworks should account for both cultural and dispositional dimensions. By addressing social norms and psychological factors—such as the consumption of certain types of pornography, sexual behaviors, and individual differences—interventions aimed at reducing the ASAM can become more targeted and effective in mitigating sexual aggression–supportive attitudes.

#### Takeaways for Possible Interventions

Based on the findings of this study, it appears necessary to address the ASAM and investigate viable solutions targeting possible associations with the porn industry and compulsive porn use. The porn industry, like other entertainment industries, may expose individuals and society to some potentially harmful stereotypes. Given the cross-sectional nature of most studies, including this study, the direction of some associations remains unclear. It is equally plausible that individuals who already endorse sexual aggression myths are more likely to be aroused by specific pornographic content (eg, humiliation or submission), rather than pornography use leading to greater ASAM. This highlights the importance of considering potential bidirectional mechanisms when exploring these associations. Indeed, while causality cannot be established, it is, however, important to find ways to remind viewers about sexual consent when promoting scenes involving such content and to promote the distinction between fantasy and reality in sexual encounters. Possible regulation mechanisms could be considered [73] for violent content but also concerning addictive porn use.

At a population level, interventions aiming to increase pornography literacy [74] and inform about the relational and affective aspects of sexual relations [75] and how to check for sexual consent can be developed and assessed, including in school-based programs [76,77], taking into consideration possible age, gender, and cultural-related issues. The association found with sexual self-esteem suggests the importance of reinforcing such a dimension within the ability to respect oneself and others’ sexual and affective needs. The promotion of sexual self-esteem (if needed) must be done with precautions, given its complex relationship with the ASAM, as shown in our findings. For instance, the promotion of sexual self-esteem can be done while emphasizing healthy sexual practices and attitudes, rather than reinforcing negative stereotypes. Finally, interventions designed to address moral incongruence might be of great importance, as they can help people resolve the cognitive dissonance associated with it and help people, especially men, reach harmony between their fantasies, porn use, and sexual behavior outside of the ASAM influence.

### Limitations and Future Studies

The study’s limitations include its cross-sectional design and a small number of people endorsing nonbinary gender. It is also limited by some single-item measurements (eg, sexual self-esteem and moral incongruence). Although we employed measurements used in prior research, we are aware of the difficulties of generalizing these results and the need for more elaborate measures in future studies.

The strengths of the data-driven approach used in this study may have been limited by the absence of certain dimensions that should be included in future research. For instance, a more refined assessment of sexual self-esteem, along with other potentially important dimensions, such as sexual function and relational interactions, could enhance the comprehensiveness of future studies.

The study introduced the assessment of different kinds of porn-arousing scenes, considering relational aspects of the contents. We cannot exclude that some wordings, such as the group scenes, may include different kinds of relational interactions. Additionally, the arousal to pornographic content was measured through participants’ self-reported responses to scene type labels (eg, humiliation, submission), without detailed descriptions or audiovisual material. This may limit the ecological validity of the measure. In addition, all participants responded to the same list of scenes, regardless of their sexual orientation, which may have affected the relevance of some items. Future research could benefit from more tailored assessments based on individual preferences.

Furthermore, given the cross-sectional design of this study, causal inferences cannot be drawn regarding the relationship between arousal to specific pornographic content and the ASAM. It remains unclear whether exposure to or preference for certain types of pornographic scenes increases the ASAM or whether individuals with higher ASAM are more likely to find such content arousing. Future longitudinal studies are needed to disentangle these directional effects and better understand the dynamics at play.

As far as we are aware, this study is the first (or one of very few) that has examined the relationships between “preferred” cyberporn scenes, sexual self-esteem, moral incongruence, and the ASAM. However, this study tested only the main effects; as such, all predictor variables were set at the same level. Future research based on this study could explore some predictor variables’ mediating and moderating effects. For instance, it would be interesting to examine if sexual coercion experiences diminish sexual self-esteem and, thus, increase the ASAM, and if the arousal to humiliation/submission cyberporn scenes is associated with potential problematic cyberporn use and the ASAM, and so forth.

### Conclusions

The regression models utilized in this study yielded important insights into the psychological and behavioral correlates of ASAM across genders. CCU, the perpetration of sexual coercion, and sexual self-esteem were found to be significant predictors of the ASAM among both male and female participants. Among men, impulsivity and moral incongruence emerged as particularly salient predictors, whereas age was a significant factor exclusively among women. Furthermore, marked gender differences were observed in the degree of arousal elicited by specific pornographic content and in the association between participants’ subjective appreciation of such content and their levels of ASAM endorsement.

The findings from this study can be used to develop interventions in the following directions: (1) gender-sensitive educational interventions focused on critical consumption of pornographic content, particularly addressing how arousal and appreciation may unconsciously reinforce acceptance of sexual aggression myths; (2) targeted interventions for reducing CCU, such as app-based self-monitoring tools and cognitive-behavioral strategies aimed at improving online sexual self-regulation; (3) given the role of moral incongruence and impulsivity in male participants, incorporate modules on ethical decision-making, impulse control, and empathy training in school- and community-based programs; (4) tailor sexual health and consent education for women by addressing developmental and age-related factors that may influence their beliefs around sexual aggression myths; (5) integrate findings into rehabilitation programs for individuals who have engaged in sexually coercive behavior, focusing on restructuring harmful beliefs, enhancing sexual self-esteem in healthy ways, and reducing impulsivity; and (6) launch broader societal campaigns to debunk sexual aggression myths and reduce their normalization, with particular attention to gender-specific messaging and outreach.

## Supplementary material

10.2196/75485 Multimedia Appendix 1 Measures.

10.2196/75485 Multimedia Appendix 2 Participants by country of residency.

## Abbreviations

AMMSA: Acceptance of Modern Myths about Sexual Aggression
ASAM: Acceptance of Sexual Aggression Myths
CCU: compulsive cyberporn use
CIUS: Compulsive Internet Use Scale
SES-P: Sexual Experience Survey–Perpetration
SES-V: Sexual Experience Survey–Victimization
UPPS-P: Urgency, Premeditation, Perseverance, Sensation Seeking, Positive Urgency
