# Supplementary material for: Predictors for Acceptance of Sexual Aggression Myths Among People Using Cyberporn: Cross-Sectional Study
Source: JMIR Form Res. 2025 Oct 16;9:e75485. doi: 10.2196/75485 (PMC12530455; doi:10.2196/75485)
Supplement: Multimedia Appendix 1 [file formative-v9-e75485-s001.pdf]

Measures

Criterion variable

Acceptance of Sexual Aggression Myths (ASAM) assessed with the Acceptance of Modern Myths about Sexual Aggression (AMMSA)

Below you will find a number of statements related to sexual violence. For each statement, please mark the number between 1 and 7 that best expresses how much you agree or disagree.

	1) I totally disagree	7) I totally agree
As long as they don't go too far, suggestive remarks and allusions simply tell a woman that she is attractive.		
It is a biological necessity for men to release sexual pressure from time to time.		
Many women tend to misinterpret a well-meant gesture as a « sexual assault ».		
Interpreting harmless gestures as ''sexual harassment'' is a popular weapon in the battle of the sexes.		
To get custody for their children, women often falsely accuse their ex-husband of a tendency toward sexual violence.		
Many women tend to exaggerate the problem of male violence.		
Women often accuse their husbands of marital rape just to retaliate for a failed relationship.		
The discussion about sexual harassment on the job has mainly resulted in many a harmless behavior being misinterpreted as harassment.		
When a woman starts a relationship with a man, she must be aware that the man will assert his right to have sex.		
A lot of women strongly complain about sexual infringements for no real reason, just to appear emancipated.		
Women like to play coy. This does not mean that they do not want sex.		

Predictor variables

Demographic data: Sex, age, sexual orientation, and relationship status

Please select your date of birth (e.g. 1980)

I am

- Man
- Woman
- Non binary

Please specify:

I am

- Celibate
- In relation, not married
- In relation, married
- Widow (er)

I am

- Heterosexual
- Homosexual
- Bisexual
- Other

Please specify:

Violent and coercive sexuality: The Sexual Experience Survey (SES)

The following questions concern sexual experiences. Place a check mark in the box showing the number of times each experience has happened. If several experiences occurred on the same occasion--for example, if one night you told some lies and had sex with someone who was drunk, you would check both boxes a and c. Since age 14 refers to your life starting on your 14th birthday until now.

1. I fondled, kissed, or rubbed up against the private areas of someone's body (lips, breast/chest, crotch or butt) or removed some of their clothes without their consent (but did not attempt sexual penetration) by:

How many times from the age of 14 until now?

	0	1	2	3+
a. Telling lies, threatening to end the relationship, threatening to spread rumors about them, making promises about the future I knew were untrue, or continually verbally pressuring them after they said they didn't want to.	○	○	○	○
b. Showing displeasure, criticizing their sexuality or attractiveness, getting angry but not using physical force after they said they didn't want to.	○	○	○	○
c. Taking advantage when they were too drunk or out of it to stop what was happening.	○	○	○	○
d. Threatening to physically harm them or someone close to them.	○	○	○	○
e. Using force, for example holding them down with my body weight, pinning their arms, or having a weapon.	○	○	○	○

2. I had oral sex with someone or had someone perform oral sex on me without their consent by:

How many times from the age of 14 until now?

	0	1	2	3+
a. Telling lies, threatening to end the relationship, threatening to spread rumors about them, making promises about the future I knew were untrue, or continually verbally pressuring them after they said they didn't want to.	○	○	○	○
b. Showing displeasure, criticizing their sexuality or attractiveness, getting angry but not using physical force after they said they didn't want to.	○	○	○	○
c. Taking advantage when they were too drunk or out of it to stop what was happening.	○	○	○	○
d. Threatening to physically harm them or someone close to them.	○	○	○	○
e. Using force, for example holding them down with my body weight, pinning their arms, or having a weapon.	○	○	○	○

3. I put my penis (men only) or I put my fingers or objects (all respondents) into a woman's vagina without her consent by:

How many times from the age of 14 until now?

	0	1	2	3+
a. Telling lies, threatening to end the relationship, threatening to spread rumors about them, making promises about the future I knew were untrue, or continually verbally pressuring them after they said they didn't want to.	○	○	○	○
b. Showing displeasure, criticizing their sexuality or attractiveness, getting angry but not using physical force after they said they didn't want to.	○	○	○	○
c. Taking advantage when they were too drunk or out of it to stop what was happening.	○	○	○	○
d. Threatening to physically harm them or someone close to them.	○	○	○	○
e. Using force, for example holding them down with my body weight, pinning their arms, or having a weapon.	○	○	○	○

4. I put in my penis (men only) or I put my fingers or objects (all respondents) into someone's butt without their consent by:

How many times from the age of 14 until now?

	0	1	2	3+
a. Telling lies, threatening to end the relationship, threatening to spread rumors about them, making promises about the future I knew were untrue, or continually verbally pressuring them after they said they didn't want to.	◌	◌	◌	◌
b. Showing displeasure, criticizing their sexuality or attractiveness, getting angry but not using physical force after they said they didn't want to.	◌	◌	◌	◌
c. Taking advantage when they were too drunk or out of it to stop what was happening.	◌	◌	◌	◌
d. Threatening to physically harm them or someone close to them.	◌	◌	◌	◌
e. Using force, for example holding them down with my body weight, pinning their arms, or having a weapon.	◌	◌	◌	◌

5. Even though it did not happen, I TRIED to have oral sex with someone or make them have oral sex with me without their consent by:

How many times from the age of 14 until now?

	0	1	2	3+
a. Telling lies, threatening to end the relationship, threatening to spread rumors about them, making promises about the future I knew were untrue, or continually verbally pressuring them after they said they didn't want to.	◌	◌	◌	◌
b. Showing displeasure, criticizing their sexuality or attractiveness, getting angry but not using physical force after they said they didn't want to.	◌	◌	◌	◌
c. Taking advantage when they were too drunk or out of it to stop what was happening.	◌	◌	◌	◌
d. Threatening to physically harm them or someone close to them.	◌	◌	◌	◌
e. Using force, for example holding them down with my body weight, pinning their arms, or having a weapon.	◌	◌	◌	◌

6. Even though it did not happen, I TRIED put in my penis (men only) or I tried to put my fingers or objects (all respondents) into a woman's vagina without their consent by:

How many times from the age of 14 until now?

	0	1	2	3+
a. Telling lies, threatening to end the relationship, threatening to spread rumors about them, making promises about the future I knew were untrue, or continually verbally pressuring them after they said they didn't want to.	○	○	○	○
b. Showing displeasure, criticizing their sexuality or attractiveness, getting angry but not using physical force after they said they didn't want to.	○	○	○	○
c. Taking advantage when they were too drunk or out of it to stop what was happening.	○	○	○	○
d. Threatening to physically harm them or someone close to them.	○	○	○	○
e. Using force, for example holding them down with my body weight, pinning their arms, or having a weapon.	○	○	○	○

7. Even though it did not happen, I TRIED to put in my penis (men only) or I tried to put my fingers or objects (all respondents) into someone's butt without their consent by:

How many times from the age of 14 until now?

	0	1	2	3+
a. Telling lies, threatening to end the relationship, threatening to spread rumors about them, making promises about the future I knew were untrue, or continually verbally pressuring them after they said they didn't want to.	○	○	○	○
b. Showing displeasure, criticizing their sexuality or attractiveness, getting angry but not using physical force after they said they didn't want to.	○	○	○	○
c. Taking advantage when they were too drunk or out of it to stop what was happening.	○	○	○	○
d. Threatening to physically harm them or someone close to them.	○	○	○	○
e. Using force, for example holding them down with my body weight, pinning their arms, or having a weapon.	○	○	○	○

8. Did you do any of the acts described in this part of the questionnaire 1 or more times?

- ○ Yes
- ○ No

If yes, what was the sex of the person or persons to whom you did them?

- Female only
- Male only
- Both females and males
- I reported no experiences

9. Do you think you may have you ever raped someone?

- Yes
- No

The following questions concern sexual experiences that you may have had that were unwanted. Place a check mark in the box showing the number of times each experience has happened to you. If several experiences occurred on the same occasion--for example, if one night someone told you some lies and had sex with you when you were drunk, you would check both boxes a and c. Since age 14 refers to your life starting on your 14th birthday until now.

1. Someone fondled, kissed, or rubbed up against the private areas of my body (lips, breast/chest, crotch or butt) or removed some of my clothes without my consent (but did not attempt sexual penetration) by:

How many times from the age of 14 until now?

	0	1	2	3+
a. Telling lies, threatening to end the relationship, threatening to spread rumors about me, making promises I knew were untrue, or continually verbally pressuring me after I said I didn't want to.	○	○	○	○
b. Showing displeasure, criticizing my sexuality or attractiveness, getting angry but not using physical force, after I said I didn't want to.	○	○	○	○
c. Taking advantage of me when I was too drunk or out of it to stop what was happening.	○	○	○	○
d. Threatening to physically harm me or someone close to me.	○	○	○	○
e. Using force, for example holding me down with their body weight, pinning my arms, or having a weapon.	○	○	○	○

2. Someone had oral sex with me or made me have oral sex with them without my consent by:

How many times from the age of 14 until now?

	0	1	2	3+
a. Telling lies, threatening to end the relationship, threatening to spread rumors about me, making promises I knew were untrue, or continually verbally pressuring me after I said I didn't want to.	○	○	○	○
b. Showing displeasure, criticizing my sexuality or attractiveness, getting angry but not using physical force, after I said I didn't want to.	○	○	○	○
c. Taking advantage of me when I was too drunk or out of it to stop what was happening.	○	○	○	○
d. Threatening to physically harm me or someone close to me.	○	○	○	○
e. Using force, for example holding me down with their body weight, pinning my arms, or having a weapon.	○	○	○	○

3. A man put his penis into my vagina, or someone inserted fingers or objects without my consent by:

How many times from the age of 14 until now?

	0	1	2	3+
a. Telling lies, threatening to end the relationship, threatening to spread rumors about me, making promises I knew were untrue, or continually verbally pressuring me after I said I didn't want to.	○	○	○	○
b. Showing displeasure, criticizing my sexuality or attractiveness, getting angry but not using physical force, after I said I didn't want to.	○	○	○	○
c. Taking advantage of me when I was too drunk or out of it to stop what was happening.	○	○	○	○
d. Threatening to physically harm me or someone close to me.	○	○	○	○
e. Using force, for example holding me down with their body weight, pinning my arms, or having a weapon.	○	○	○	○

4. A man put his penis into my butt, or someone inserted fingers or objects without my consent by:

How many times from the age of 14 until now?

	0	1	2	3+
a. Telling lies, threatening to end the relationship, threatening to spread rumors about me, making promises I knew were untrue, or continually verbally pressuring me after I said I didn’t want to.	○	○	○	○
b. Showing displeasure, criticizing my sexuality or attractiveness, getting angry but not using physical force, after I said I didn’t want to.	○	○	○	○
c. Taking advantage of me when I was too drunk or out of it to stop what was happening.	○	○	○	○
d. Threatening to physically harm me or someone close to me.	○	○	○	○
e. Using force, for example holding me down with their body weight, pinning my arms, or having a weapon.	○	○	○	○

5. Even though it didn’t happen, someone TRIED to have oral sex with me, or make me have oral sex with them without my consent by:

How many times from the age of 14 until now?

	0	1	2	3+
a. Telling lies, threatening to end the relationship, threatening to spread rumors about me, making promises I knew were untrue, or continually verbally pressuring me after I said I didn’t want to.	○	○	○	○
b. Showing displeasure, criticizing my sexuality or attractiveness, getting angry but not using physical force, after I said I didn’t want to.	○	○	○	○
c. Taking advantage of me when I was too drunk or out of it to stop what was happening.	○	○	○	○
d. Threatening to physically harm me or someone close to me.	○	○	○	○
e. Using force, for example holding me down with their body weight, pinning my arms, or having a weapon.	○	○	○	○

6. Even though it didn’t happen, a man TRIED to put his penis into my vagina, or someone tried to stick in fingers or objects without my consent by:

How many times from the age of 14 until now?

	0	1	2	3+
a. Telling lies, threatening to end the relationship, threatening to spread rumors about me, making promises I knew were untrue, or continually verbally pressuring me after I said I didn't want to.	○	○	○	○
b. Showing displeasure, criticizing my sexuality or attractiveness, getting angry but not using physical force, after I said I didn't want to.	○	○	○	○
c. Taking advantage of me when I was too drunk or out of it to stop what was happening.	○	○	○	○
d. Threatening to physically harm me or someone close to me.	○	○	○	○
e. Using force, for example holding me down with their body weight, pinning my arms, or having a weapon.	○	○	○	○

7. Even though it didn't happen, a man TRIED to put his penis into my butt, or someone tried to stick in objects or fingers without my consent by:

How many times from the age of 14 until now?

	0	1	2	3+
a. Telling lies, threatening to end the relationship, threatening to spread rumors about me, making promises I knew were untrue, or continually verbally pressuring me after I said I didn't want to.	○	○	○	○
b. Showing displeasure, criticizing my sexuality or attractiveness, getting angry but not using physical force, after I said I didn't want to.	○	○	○	○
c. Taking advantage of me when I was too drunk or out of it to stop what was happening.	○	○	○	○
d. Threatening to physically harm me or someone close to me.	○	○	○	○
e. Using force, for example holding me down with their body weight, pinning my arms, or having a weapon.	○	○	○	○

8. Did any of the experiences described in this part of the questionnaire happen to you 1 or more times?

○ Yes

○ No

If yes, what was the sex of the person or persons who did them to you?

- ○ Female only
- ○ Male only
- ○ Both females and males
- ○ I reported no experiences

---

9. Have you ever been raped?

- ○ Yes
- ○ No

Compulsive Cyberporn Use (CCU) assessed with the Compulsive Internet Use Scale (CIUS)

In the following questions, by the " internet" we understand "PORNOGRAPHIC WEBSITES". Answers can be given on a 5-point scale.
Thank you for the respond to each statement, to indicate how much you agree or disagree with the statement During the LAST YEAR...

	Never	Seldom	Sometimes	Often	Very often
How often do you find it difficult to stop using the internet when you are online?	○	○	○	○	○
How often do you prefer to use the internet instead of spending time with others (e.g. partner, children, parents, friends)?	○	○	○	○	○
How often are you short of sleep because of the internet?	○	○	○	○	○
How often do you look forward to your next internet session?	○	○	○	○	○
How often have you unsuccessfully tried to spend less time on the internet?	○	○	○	○	○
How often do you neglect your daily obligations (work, school, or family life) because you prefer to go on the internet?	○	○	○	○	○
How often do you go on the internet when you are feeling down?	○	○	○	○	○
How often do you feel restless, frustrated, or irritated when you cannot use the internet?	○	○	○	○	○

Arousing cyberporn scenes

The following pornographic scenes are:

	Very arousing	Not arousing at all
Submission		
Humiliation		
Soft		
Groups with many males		
Groups with many females		

Moral incongruence

I believe that pornography use is morally wrong

1- Strongly disagree — 7- Strongly agree

Impulsivity measured with the Short UPPS-P Impulsive Behavior Scale

Below are numbers of statements that describe ways in which people act and think.
For each statement, please indicate how much you agree or disagree with the statement. Be sure to indicate your agreement or disagreement for every statement below

	Agree Strongly	Agree	Disagree	Disagree Strongly
When I'm really excited, I tend not to think about the consequences of my actions.	○	○	○	○
When I am upset I often act without thinking	○	○	○	○
In the heat of an argument, I will often say things that I later regret	○	○	○	○
When overjoyed, I feel like I can't stop myself from going overboard	○	○	○	○
I often make matters worse because I act without thinking when I am upset	○	○	○	○
I tend to act without thinking when I am really excited	○	○	○	○
When I feel rejected, I will often say things that I later regret	○	○	○	○
When I'm very happy I feel like it is OK to give in to cravings and overindulge	○	○	○	○

Sexual self-esteem

Indicate how much you agree or disagree with the statement below

	Not very true of me	Not true	True	Very true of me
I have high sexual self – esteem	○	○	○	○
