# Supplementary material for: Predictors for Acceptance of Sexual Aggression Myths Among People Using Cyberporn: Cross-Sectional Study
Source: JMIR Form Res. 2025 Oct 16;9:e75485. doi: 10.2196/75485 (PMC12530455; doi:10.2196/75485)
Supplement: Multimedia Appendix 2 [file formative-v9-e75485-s002.docx]

# Appendix

**Participants by country of residency**

| Place of residence (among 47 distinct nationalities) | N | % |
| --- | --- | --- |
| Australia | 25 | 1.4 |
| Belgium | 1 | .1 |
| Canada | 78 | 4.4 |
| Chile | 3 | .2 |
| Czech Republic | 1 | .1 |
| Estonia | 2 | .1 |
| Hungary | 5 | .3 |
| Ireland | 22 | 1.2 |
| Italy | 8 | .5 |
| Korea | 1 | .1 |
| Latvia | 1 | .1 |
| Mexico | 6 | .3 |
| Netherlands | 6 | .3 |
| New Zealand | 15 | .8 |
| Poland | 12 | .7 |
| Portugal | 8 | .5 |
| Slovenia | 3 | .2 |
| South Africa | 116 | 6.5 |
| Spain | 6 | .3 |
| Sweden | 1 | .1 |
| Switzerland | 2 | .1 |
| United Kingdom | 807 | 45.6 |
| United States | 461 | 26.0 |

*Note:* This table is based on 1590 participants who listed their countries and nationalities.
